# Prevalence and characteristics of depressive disorders in early adolescents in central Norway

**DOI:** 10.1186/1753-2000-5-28

**Published:** 2011-08-31

**Authors:** Anne Mari Sund, Bo Larsson, Lars Wichstrøm

**Affiliations:** 1 Department of Neuroscience, Faculty of Medicine, Norwegian University of Science and Technology, N- 7489, Trondheim, Norway; 2 St.Olav's University Hospital, Trondheim, Norway; 3 Department of Psychology, Norwegian University of Science and Technology, N- 7491, Trondheim, Norway

**Keywords:** Depressive disorders, MDD, Dysthymia, Depression NOS, adolescence, epidemiology, health service

## Abstract

**Background:**

Prevalence of depressive disorders among adolescents has varied across studies. The present study aims to assess current and lifetime prevalence and characteristics of adolescent Major Depressive Disorder (MDD), Dysthymia and Depression NOS among adolescents in Central Norway in addition to socio-demographics and use of mental health care.

**Method:**

In the Youth and Mental Health Study a representative sample of 2432 junior high school students (mean age 14.9 years, SD = 0.6) from two counties in Central Norway were screened with the Mood and Feelings Questionnaire (MFQ). A subset of 345 of these adolescents (72.5% girls), 220 high scorers (MFQ = > 26), 74 middle scorers (MFQ 7-25), and 50 low scorers (MFQ < 7), 1 unknown score, were drawn and interviewed with the Kiddie SADS-PL (Present-Life Version). In all, 79% had parental interviews as well. All estimates of prevalence rates and population shares were weighted back using a sandwich estimator to yield true population estimates.

**Results:**

Almost one in four subjects (23%) had life-time depression. Prevalences of current Major Depressive Disorder (MDD), Dysthymia and "Double depression" were 2.6%, 1.0% and 0.6%, respectively, and for Depression NOS 6.3%.

All depressive disorders were characterized by long duration of episodes with large variations, and for any depressive disorder onset before 12 years of age. In multivariate analyses MDD and Dysthymia were most strongly associated with gender and not living with both biological parents. There was no gender difference for Depression NOS. Although a considerable number of depressed subjects had received mental health care, the reason for contact with services was seldom due to affective symptoms. Less than 20% had been in contact with specialist mental health services.

**Conclusion:**

High rates of Depression NOS, early onset of depressive episodes, long duration, and low use of specialized services point to the need for improved diagnostic assessment and treatment for young individuals.

## Background

Depression leads to suffering and disability among adolescents [1], and also has serious long-term consequences persisting into young adulthood [2-4].

It is well known that pre-adolescent depression is rare with no gender difference [5], and that the prevalence rate increases sharply from early adolescence [6,7] with a preponderance among girls [8].

However, both prevalence rates and the size of the gender difference vary between studies. In Europe, the prevalence of major depression registered ranges from a 1-year prevalence among 14 -17-year olds of 3.4% in Germany [9] and of 16-17-year olds of 5.8% in Sweden [10] to a 6-month prevalence of 1.9% among 15-year-olds in the UK [11], 2.7% among 13-18-year olds in the Netherlands [12] and 5.0% among 13-15-year olds in Switzerland [13].

In a recent meta-analysis the prevalence of depression (MDD and/or Dysthymia) was 5.7% among 13-18- year olds with a female to male ratio of 1.3:1 [14]. While the majority of the studies included in this meta-analysis were from the USA, in Europe [9-13] the point-prevalence figures seldom exceeded the grand mean in this review. Thus, the possibility of lower rates of depressive disorders in Europe ought to be explored further by including data from other sites. Further, reported rates by gender tend to reveal a greater difference in prevalence rates in European surveys. For example, in Germany, a girls/boys ratio of 1.9:1 was found [9], but in Sweden [10] and in Switzerland [13], the corresponding rates were 4.1:1 and 8.9:1, respectively. Thus, the lower rates of adolescent depression in Europe may reflect lower rates among boys.

While differences in prevalence rates may reflect true rates, they may also be related to differences in methods and measures used for assessing depression, sampling procedures, time frame, age, information source and the type of depressive disorder assessed. It should be noted that prevalence of minor depression is seldom evaluated. Similarly, age of onset and other characteristics of the depressive episode for Dysthymia and Depression NOS in particular, are understudied in general populations and across countries and cultures [15]. Further, to date findings on the relationships between socio-demographics, ethnicity and depression in children and adolescents are not conclusive. Concern has also been raised in Europe [16] and in the USA [17] as to whether depressed adolescents in the general population have access to and receive proper mental health care. Also, since there still are inconsistencies between studies, more research is needed comparing results from various countries [15].

In the pursuit of evidence regarding regional prevalence rates and gender differences we provide data from a representative, community study of Norwegian adolescents in Central Norway. Our first aim was to estimate current (2-month) and life-time prevalence rates of various depressive disorders comprising Major Depressive Disorder (MDD), Dysthymia and Depression NOS, among 14-16-year-old adolescents using a 2-stage strategy including screening and subsequent interviews with adolescents and parents. A second aim was to examine onset, duration and severity of these disorders and examine their relationships to socio-demographics. Lastly, we report on the use of mental health care as reported by adolescents and parents. Knowledge on the prevalence, gender differences and characteristics of various depressive disorders like onset age, and help-seeking behavior, will enable us to recognize depression among youth earlier and help to initiate appropriate interventions.

## Methods

### Sampling and participants

**Figure 1:**
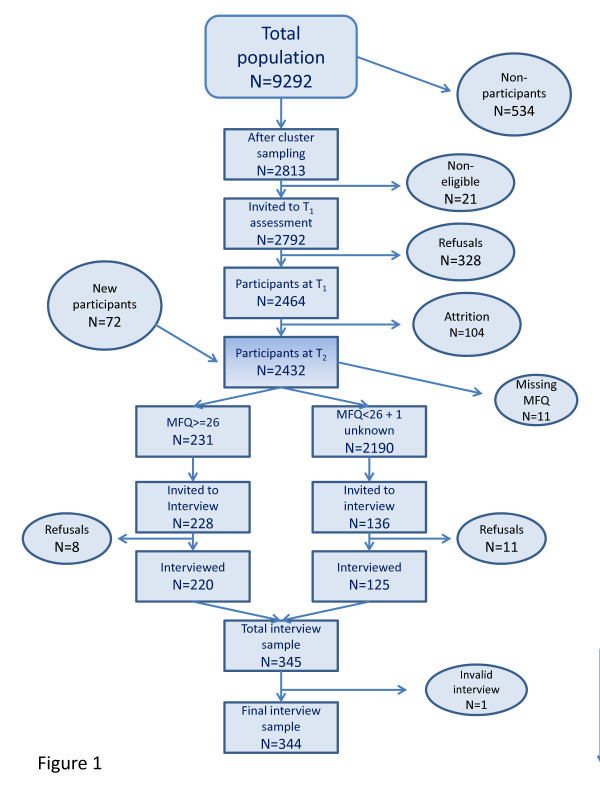
**Flow-chart of the Youth and Mental Health Study in Central Norway**. The flow-chart shows the numbers of participants in the various stages in the project.

The Youth and Mental Health Study is a longitudinal study of depressive symptoms and disorders among adolescents in two counties in Central Norway (South and North-Trøndelag), that started when the adolescents were 12-15-years old (8th and 9th grade in Norway). At the time, these areas comprised a population of 390 000 inhabitants, including one city, Trondheim, with 146 000 inhabitants (the third largest in Norway). The total population in the selected age group comprised 9292 pupils attending public (98.5%) or private schools in autumn 1998, while 38 pupils attending special schools were not included in this number. Students from very small schools in very remote areas comprising 534 adolescents did not participate due to logistical considerations. The sample was stratified according to urbanity and geography. A cluster sampling method was chosen using schools as sampling units. Schools were drawn with a probability according to size (proportional allocation) within each stratum. A total of 2792 subjects were eligible for the study [18]. See Flow Chart, Figure 1.

In September 1998, at T_1_, the first assessment with questionnaires was performed with a participation rate of 88.3%. The final sample of 2464 students (50.8% girls) from 22 schools was stratified as follows: (1) City of Trondheim (n = 484, 19.5%; (2) Suburbs of Trondheim (n = 432, 17.5%); (3) Coastal region (n = 405, 16.4%); and (4) Inland region (n = 1143, 46.4%). The mean age was 13.7 years (range 12.5-15.7; SD 0.58). The non-responders (n = 328) were significantly more often boys [χ ^2 ^(1) = 22.11, p < 0.001] and younger adolescents [χ ^2 ^(1) = 5.56, p < 0.05].

The same students were contacted one year later (T_2_, 9^th ^and 10^th ^grade) and reassessed (N = 2432) at a mean age of 14.9 years (range 13.7-17.0, SD0.59). Attrition from T_1 _to T_2 _was 4.3% (N = 104). The non-participants at T_2_, had higher mean total MFQ (see below) scores at T_1 _[17.3 vs. 10.4, t (2442) = 7.13, p < 0.001], and more often had a non-Norwegian background [χ ^2 ^= 13.45 (1), p < 0.001]. No gender, grade or SES differences between the two groups were found. In addition, at T_2_, 72 students from the original sample who were invited to participate at T_1_, and then denied participating, consented to participate at T_2 _(51.3% boys).

To assess the generalizability of our findings, data from three large representative nationwide surveys of depressive mood in Norwegian adolescents conducted in 1992, 2002 and 2010 were used [19]. Adolescents (aged 13 to 19 years) in the Central Norwegian counties scored slightly below (.13 standard deviations) those in the remaining parts of Norway on the Depressive Mood Inventory [20] [1.73 vs. 1, 81, t (30,939) = 7.65, p < 0.001].

### Procedures

#### Questionnaires

At T_1 _and T_2 _all the students completed identical questionnaires during two school hours. In the present study, information collected at T_2 _was used on demographics and levels of depressive symptoms, using the Mood and Feelings Questionnaire (MFQ)[21]. Twenty-six students had missing data on gender.

#### Interviews

At T_2_, participants were invited for the interview phase based on their MFQ scores. The data collection at T_2 _lasted 5 months due to the intervening interviewing. All students with high depression symptom scores (MFQ = > 26) defined as high scorers were invited for interview. For every two high scorers, one low scoring (MFQ < 7) or middle scoring (MFQ = 7 - 25) adolescent of the same sex and school class was randomly selected. We aimed to employ the conventional definition of high scorers including those scoring higher than the 90^th ^percentile score on the MFQ. In the present sample, at T_1_, the 90^th ^percentile score on the MFQ was 24 [22]. At T_2_, the primarily results from the first schools indicated an expected rise, although small, in depressive symptom levels during the preceding year. We therefore decided to raise the cut-off to MFQ = 26, which was reached by 9.5% of the final study sample. The low/middle cut-off score was based on a split-half on the MFQ for the remaining subjects. Eleven students were removed from the interview selection process because of missing MFQ data.

Of all invited students (N = 364), 94.8% (N = 345) completed the interview (72.5% girls, with a mean age of 15.0 years (range 13.8 - 16.6 years, SD = 0.6). Erroneously, three individuals were not invited for interview. Eight of the high-scorers and 11 of the low/middle scorers refused to participate. One interview was lacking MFQ data (See Table 1 for demographic information on the interview sample and Table 2 for an overview of the selection process).

**Table 1:** Demographics of the interview sample (N = 345: 220 high scoring individuals on the Mood and Feelings Questionnaire, i.e. MFQ > = 26 and 124 medium and low scorers, i.e. MFQ < 26: 1 missing on the MFQ) from a representative sample of 2432 adolescents from Central Norway, with a mean age of 15 years

	N	Percentage
Gender		
1. Girls	250	72.50%
2. Boys	95	27.50%

Geography		
1. Inner city	66	19.10%
2. Suburbs	66	19.10%
3. Coast	51	14.80%
4. Inland	162	47.00%

Parental socioeconomic status		
1. Professional leader (upper class)	33	9.60%
2. Upper middle class	85	24.60%
3. Lower middle class	53	15.40%
4. Primary industry	27	7.80%
5. Manual worker	142	41.20%
6. No information	5	1.40%

Ethnicity		
1. Both parents Norwegian	316	91.60%
2. One parent Norwegian	9	2.60%
3. Both parents East-European	11	3.20%
4. Both parents non-Western/non European	9	2.60%

Living arrangement		
1. Living with both parents	221	64.10%
2. Living with mother	47	13.60%
3. Living with father	15	4.30%
4. With one parent and stepparent	47	13.60%
5. Sharing time between parents	11	3.20%
6. Living with grandparents/foster parents	4	1.20%

**Table 2:** Numbers in the T2 population sample (N = 2432)* and numbers being interviewed by MFQ group and by gender (N = 344), percentages shown

MFQ group**	MFQ 0-6		MFQ 7-25		MFQ > = 26	
	**All**	**Interviewed (%)**	**All**	**Interviewed (%)**	**All**	**Interviewed (%)**

Girls	384	37 (9.6%)	659	53 (8%)	171	160 (93.6%)

Boys	669	13 (1.9%)	452	21 (4.7%)	60	60 (100%)

All	1053	50 (4.8%)	1111	74 (6.7%)	231	220 (95.2%)

* 26 missing by gender and 11 by MFQ

** one interview lacked MFQ

Of the adolescents, 79.4% had at least one parent as a separate informant. Diagnostic assessment was based on interview data obtained from both adolescents and parents, and on adolescent report only when no parent was available. The mean time elapsed between the completion of the MFQ and the interviews was 20 days (range 1 - 164 days, SD 17.1) for the adolescents, while for the parents the mean interval was 24 days (range 0 - 164 days, SD = 18.3). Of the sample, 91% of adolescents were interviewed approximately within one month. All interviews were conducted at the student's own school by five trained interviewers (further details of the sampling and methods can be obtained from the first author).

### Assessment

#### Sociodemographics

Parent Socio-economic status (SES) was measured by classifying mothers' and fathers' occupations using the ISCO-88 [23]. Information on parents' occupation was collected during the interview with the parent (s). Only when no parent was interviewed, information from the adolescents was used. Coding of parent occupation was then based on two open-ended questions posed to the adolescent: "What occupation does your father/mother have?" and "What does he/she do at work?" The responses were classified in 5 groups ranging from professional leader/upper class to manual workers. Perceived economy was assessed by asking the parents to assess the economy in the family on a 5-point scale ranging from "Very satisfied" to "Very dissatisfied".

Household composition was dichotomized as follows; whether the young person lived with both biological or adoptive parents, or not. Parents' country of origin was used to classify ethnic background in regard to having one or both parents born in Norway or not. Six adopted adolescents were classified as native Norwegians.

#### Use of mental health care

Both adolescents and their parents were asked open-ended questions as to whether the adolescents had ever received help because of mental health problems, problem type, and who provided help. The date and reason for each contact were recorded. All community and specialist health service contacts as well as contacts with professionals at schools and child protection services and others, were recorded. Categories in the present study were defined by the researchers after completion of the interviews. In the multivariate analyses all information was collapsed into two variables representing current and previous mental health care.

#### The Mood and Feelings Questionnaire(MFQ) [21]

This 34 - item rating scale for children and adolescents aged 8 - 18 years was developed to cover all the DSM-IV symptoms of major depressive disorder (MDD) [24]. The last item of the MFQ parent version was included in the MFQ in the present study: "I was not as happy as usual when praised or rewarded". The individual is asked to report his or her feelings for the preceding two weeks on a 0-2 scale (0 = "Not true", 1 = "Sometimes true", 2 = "True"), and the total sum score ranges between 0 and 68. The instrument has been used to screen for depressive symptoms among adolescents in clinical samples [25,26] and the general population [27]. For the present study, the MFQ was translated and back - translated and approved by the originator (dr. Angold). In the original school sample at T_1_, test-retests for 3 weeks and 2 months were found to be 0.84 and 0.80, respectively. The internal consistency α = 0.91 [22] and convergent validity with the Beck Depression Inventory(BDI) [28] was r = 0.90.

#### Kiddie -SADS PL (Present-Life version)

This well established semi-structured diagnostic interview [29] was used to assess present and past episodes of psychopathology in children and adolescents and based on DSM-IV Axis I criteria (American Psychiatric Association)[24]. Each individual symptom is rated on a 0-3 scale with a score of 3 representing clinical threshold. It allows probing and includes a screening interview with 5 supplements and impairment ratings, both globally (C-GAS)[30] and for every diagnosis. Validity and reliability data have been reported [29]. High agreement between interviewers, good to excellent test-retest reliability, and high concurrent validity with the BDI and parent rating on the Child Behavior Checklist (CBCL)[31] internalizing problem scale has been found [29]. A diagnostic criterion could be met by endorsement of symptom presence by either the adolescent or the parent(s), based on empirical evidence that both the child and the parent add unique and valid information to the diagnosis [32]. Co-morbidity with depression was also assessed in the interview, but is not presented in the present study. The current time frame for ascertainment of depressive diagnoses was 2 months, i.e. included depressive diagnoses occurring within the last 2 months. The worst previous episode (WPE) of depression was also recorded. For an episode to be considered *previous*, the adolescent should have had a minimum of two months free from symptoms. Onset and duration data were collected both for current and previous episodes.

#### Depressive disorders

*Major depressive disorder *(MDD) was defined as an adolescent having 5 of 9 DSM-related symptoms, one being depressed mood/irritability or anhedonia, at the threshold level for at least 14 days. *Dysthymia *was defined as having 3 of 7 symptoms, one being depressed mood/irritability lasting at least one year. "*Double depression*," i.e. the existence of both MDD and Dysthymia, was present when Dysthymia preceded the MDD episode for at least one year. In order to receive a diagnosis of MDD or Dysthymia, the adolescent also had to have a C-GAS score below 71 or impairment in one of the three areas: family, school, or friendship, as assessed in the interview. A diagnosis of *Depression NOS *was defined according to the DSM-IV -TR criterion [33] as having 2 - 4 out of 9 symptoms, including at least one of the main criteria, for 14 days or longer. *Short Brief Recurrent Depression *was defined as a recurrent episode of at least 5 of 9 symptoms lasting less than 2 weeks. It was found in 3 adolescents who were included in the Depression NOS group. Both current diagnoses and at least one WPE episode were recorded to estimate life-time prevalence.

#### Interrater reliability

Before interviewing, agreement was checked for all interviewers against the project leader (AMS) as the "golden standard". For Kiddie-SADS-PL screening symptoms Kappa was 0.71 (range 0.66 - 0.74), and for all affective symptoms Kappa was 0.75 (range 0.75 - 0.82). To minimize interview drift, the supervisor (first author) met regularly with the interviewers both individually and as a group. Halfway into the study, Kappa coefficients were re-calculated: for all screening symptoms Kappa was 0.83 (range 0.77- 0.89) and for all affective symptoms, 0.82 (range 0.79 - 0.92).

### Ethics

The study was approved by the Regional Committee for Medical Research Ethics as well as by local school authorities in the two counties and the school boards. Written consent was obtained both from the adolescents and the parents based on standards prescribed by the Norwegian Data Inspectorate. Both parents and adolescents were offered confidentiality except for acute emergency situations. Depending on symptom severity, an adolescent could be offered a referral to the school health nurse, the local GP, or the nearest child and adolescent psychiatric outpatient unit. While 28 adolescents (8.1%) were offered a referral, only 9 (2.6%) accepted one.

### Statistics

Statistical analyses were performed using PASW Statistics 18.0 software (SPSS, Inc., Somers, NY, USA). All estimates of prevalence rates and estimated population shares were calculated using the Huber-White sandwich estimator taking into account the inclusion probability for each stratum [34]. Hence true general population estimates are presented.

Relationships between categorical variables were analyzed using chi-square statistics.

Based on earlier research findings that these disorders have similar characteristics [35], MDD and Dysthymia were grouped together to form a severe depression group when analyzing the various demographic correlates. Associations between depression diagnostic subgroups and various demographic correlates such as gender, ethnicity, living situation, parental SES and region, were analyzed with both univariate and multivariate multinomal logistic regression analyses with crude and adjusted ORs and 95% CI, also taking sampling weight into consideration. In order to control for number of multiple tests, an incremental Bonferroni correction was applied. When nominal scales with more than two values were analyzed, a Sequential Sidak procedure was used for significance testing.

## Results

### Prevalence of depressive disorders

As can be seen in table 3, 94 adolescents received a diagnosis of current depression, corresponding to a weighted prevalence estimate of 9.4% for any current depression in the general population. The majority of the disorders consisted of Depression NOS (6.3%), whereas the 2-month or current prevalences of MDD and Dysthymia were 2.6% and 1.0%, respectively. As shown in table 2, 159 adolescents (23.0%) had suffered from some kind of a depressive disorder during their life time (minimum figures) consisting of a current depressive episode or an earlier episode (WPE) or both. This figure indicates that almost one in four in this population at some time point had qualified for a depressive diagnosis. Currently, in weighted analyses, "Double depression" was found in 0.6% (95% CI 0.5-0.7) (unweighted n = 12), and life-time "Double depression" in 1.2% (95% CI 0.5-2.7) (unweighted n = 16) of the sample.

**Table 3:** Estimated current (last 2-month) and lifetime prevalence of depressive disorders (with 95% CI) and gender differences among adolescents in Central Norway (weighted analyses)

	All		Girls		Boys		Gender difference	
	**unweighted n's)**	**Estimate (95%CI)**	**unweighted n's)**	**Estimate (95% CI)**	**unweighted n's)**	**Estimate (95% CI)**	**Chi-sq. (df)**	**p-value**

*Current*								

MDD	36	2.6 (1.5 - 4.4)	31	4.7 (2.6 - 8.4)	5	0.4 (0.3 - 0.6)	6.22 (1)	< 0.001

Dysthymia	21	1.0 (0.8 - 1.1)	18	1.7 (1.4 - 2.0)	3	0.3 (0.2 - 0.4)	1.75 (1)	< 0.001

Depression NOS	49	6.3 (3.7 - 10.3)	40	8.2 (4.9-13.3)	9	4.3 (1.4-12.8)	2.21 (1)	NS

Any depression	94	9.3 (6.4 - 13.3)	79	13.7 (9.5-19.2)	15	5.2 (1.7-12.8)	7.96 (1)	0.04

*Lifetime*								

MDD	64	5.8 (3.9 - 8.6)	55	10.7 (6.9 - 16.1)	9	0.8 (0.7 - 1.0)	15.38 (1)	< 0.001

Dysthymia	39	5.4 (3.2 - 9.1)	33	8.5 (4.9 - 14.1)	6	2.3 (0.5-10.2)	6.42 (1)	NS

Depression NOS	72	13.0 (9.0- 18.4)	56	14.1 (9.4-20.6)	16	11.9 (6.2-21.6)	0.38 (1)	NS

Any depression	159	23.0 (18.1 - 28.8)	130	31.1(24.3-38.7)	29	14.8 (8.5-24.4)	12.81 (1)	< 0.01

Twenty-four adolescents had a depressive episode both currently and previously. Of 16 adolescents with a current severe depression(= MDD and/or Dysthymi), 9 had had a previous severe depression and 7 a previous Depression NOS. While among 8 individuals with a current Depression NOS, 4 had had a severe and 4 a Depression NOS previously.

Gender differences were pronounced with an estimated 2-month prevalence for any depressive disorder of 13.7% (95% CI 9.5 - 19.2) among girls compared to 5.2% (95% CI 1.7-12.8) among boys (p < 0.05). The gender difference (see table 3) in rates of current MDD and Dysthymia was larger than for Depression NOS in which no such gender difference was found for current or lifetime disorder.

### Duration

As shown in table 4, mean duration of current MDD episodes was longer than episodes of Depression NOS, which on the contrary had longer WPE episodes. A current diagnosis of Dysthymia had the longest mean duration, approximately 3.5 years while WPE Dysthymia lasted around 1.5 years, similar to the duration of WPE Depression NOS.

**Table 4:** Estimated mean duration, age of onset and C-GAS levels (with 95% CI's) for current and worst previous episode (WPE) of depressive disorders among adolescents in Central Norway (weighted analyses)

	Duration in months			
	***Current episode***		***Worst previous episode***	

	**Estimate (95% CI)**	**S.E**.	**Estimate (95% CI)**	**S.E**.

MDD	10.60 (6.46- 14.74)	2.04	10.67 (4.59-16.76)	2.96

Dysthymia	43.51 (32.84- 54.17)	5.09	19.36(15.52-23.21)	1.80

MDD + Dysthymia*	10.13 (6.36- 13.89)	1.69	4.09 (0.78-7.38)	0.77

Depression NOS	8.30 (6.04-10.57)	1.11	21.20 (4.67-37.74)	8.04

Any Depressive Disorder	14.22 (12.14 - 16.30)	1.05	16.57(9.82 - 23.31)	3.40

	**Age in years when episode started**			

	***Current episode***		***Worst previous episode***	

	**Estimate (95% CI)**	**S.E**.	**Estimate (95% CI)**	**S.E**.

MDD	14.05 (13.431-14.79)	0.36	13.30 (12.60-14.00)	0.34

Dysthymia	11.46 (10.55-12.40)	0.44	10.88 (10.16-11.60)	0.34

MDD + Dysthymia*	14.22 (13.72-14.73)	0.22	14.50 (14.17-14.83)	0.77

Depression NOS	14.00 (13.68 - 14.33)	0.16	10.53 (9.14-11.93)	0.68

Any Depressive Disorder	14.06 (13.78 - 14.33)	0.14	11.28 (10.53-12.03)	0.38

	**C-GAS level**			

	***Current episode***		***Worst previous episode***	

	**Estimate (95% CI)**	**S.E**.	**Estimate (95% CI)**	**S.E**.

No depressive disorder	84.27(82.65-85.89)	0.82	75.91(69.30 - 82.52)	3.33

MDD	56.95(53.49-60.42)	1.76	56.65 (53.64 - 59.6)	1.52

Dysthymia	69.66 (67.76-71.55)	0.97	59.77 (54.52 - 65.01)	2.65

MDD + Dysthymia	55.99 (54.62-57.36)	0.70	59.51(53.60 - 65.42)	2.99

Depression NOS	70.13(65.58-74.69)	2.31	61.26 (55.98 - 66.54)	2.67

Any Depressive Disorder	66.34 (62.11 - 70.56)	2.15	59.99 (56.78 - 63.20)	1.62

* note: Duration and age of onset of "Double Depression" starts with a MDD episode when Dysthymia has lasted a minimum of one year

### Age of onset

Current episodes of both MDD and Dysthymia started about 9 months later than for WPE episodes. With respect to WPE, both Dysthymia and Depression NOS had an earlier mean onset than MDD. "Double depression" had the latest onset, not being recorded before a dysthymic episode had lasted one year according to the DSM-IV criteria (see table 4).

### Functional level

Adolescents having a current or previous MDD or a current "Double depression" showed the lowest functioning levels on the C-GAS (see Table 4). Although adolescents with current Depression NOS showed the highest functioning levels, they still had C-GAS scores 14 points lower than those without any depressive disorder. It should be noted that adolescents with WPE diagnoses of Dysthymia or Depression NOS had C-GAS scores about 10 points lower than for those with a current diagnosis (see Table 4).

### Demographics

Associations between demographics and current severe depression (MDD, Dysthymia and "Double Depression") and Depression NOS are shown in table 5. Severe depression and depression NOS were separately contrasted to no depression and severe depression to Depression NOS. When comparisons with no depression were made, while controlling for demographics and geography, the results from multivariate adjusted regression analyses showed that gender and not living with both biological parents were associated with current severe depression, but not with Depression NOS. The latter disorder, however, was associated with adolescents living in the suburbs. Further, severe depression was more strongly associated with parents working in primary industry than with higher socio-economic class, and with inland areas as opposed to urban settlements, in comparison with no depression. For Depression NOS, a lowered risk emerged for adolescents in the lower middle class compared to those with severe depression and no depression. The OR's of severe depression versus Depression NOS are shown with symbols in table 5, and can be read out of the table by dividing one OR with the other. The findings of adjusted analyses showed that gender, not living with biological parents and belonging to the lower middle class were more strongly associated with severe depression than depression NOS. No effects of perceived economy or ethnicity were found.

**Table 5:** Crude and adjusted Odds ratios (95% Confidence Intervals) with age and ethnicity as covariates for current/2-month severe depression (= MDD and/or Dysthymia) and Depression NOS versus no depression and severe depression versus Depression NOS with regard to demographics among adolescents in Central Norway (weighted analyses)

	*Current (last 2 months)*			
	**Severe depressions**		**Depression NOS**	

	**Crude**	**Adjusted**	**Crude**	**Adjusted**

Gender (reference category = male)	11.42A¶ (6.38-20.44)	9.15***¶ (4.46-18.79)	2.10 (0.57-7.76)	2.38 (0.83-6.86)

Living situation (reference category = living together with both biological parents)	4.34A¶ (1.89-9.98)	4.55**¶ (1.75-11.84)	0.90 (0.30-2.73)	1.12 (0.40-3.16)

Parental SES (reference category = upper class)				

Upper middle class	1.98 (0.05-7.11)	1.89 (0.46-7.73)	0.40(0.39 - 4.24)	0.48 (0.05-4.26)

Lower middle class	2.75 ¶ (1.12-6.75)	1.22¶ ¶ (0.38-3.94)	0.09A (0.01 - 0.71)	0.05*** (0.01-0.46)

Primary industry	4.04A (1.14-14.3)	6.13* (1.65-22.85)	1.27 (0.09 - 18.91)	1.06 (0.09 - 12.91)

Manual workers	2.49 (0.67-8.9)	2.14 (0.50-9.30)	1.09 (0.14 - 8.5)	0.82 (0.12 -5.22)

Region (reference category = old town)				

Suburban areas	1.07¶ (0.51- 2.27)	1.83 (0.67 - 5.04)	6.00 A (1.58 - 22.86)	7.38*** (1.77 - 30.77)

Coast	0.90¶ (0.39-2.01)	0.87 (0.32 - 2.33)	6.74 A (1.79 - 25.36)	5.48 (1.11- 27.13)

Inland	2.02 (0.80-5.14)	3.08* (1.01-9.35)	2.65 A (1.04 - 6.77)	4.61 (1.10 - 6.23)

Note: A = significant according to an incremental Bonferroni correction. * p < 0.05, ** p < 0.01, *** p < 0.001 according to a Sequential Sidak procedure. Severe depressions (MDD and/or Dysthymia) significantly different from Depression NOS: ¶ p < 0.05, ¶¶ p < 0.01 according to a Sequential Sidak procedure.

### Use of mental health care

The estimated percentages of adolescents in the population sample who had been in contact with helping agencies because of mental health problems, either currently or lifetime in the different diagnostic depression groups are presented in table 6. Out of 159 adolescents having had any lifetime depressive disorder, 87 adolescents, or an estimated population share of 48.2%, had received mental health care in their life-time. A majority of adolescents with MDD had received help, regardless of having a current MDD or life-time MDD. The results of logistic regression analyses showed that all current depressive disorders increased the probability of receiving current mental health care (MDD OR 206.80, 95% CI 58.60-729.80; Dysthymia OR 6.86 95% CI 2.02-23.3; "Double depression" OR 27.7 95%CI 10.28-74.70 and Depression NOS OR 5.83 95%CI 2.00-16.96). Lifetime depression was also associated having received mental health care during lifetime (MDD OR 35.79, 95%CI 8.89-144.06; Dysthymia OR 8.36, 95%CI 1.57-44.57; "Double depression" OR 49.56, 95%CI 12.34-199.02 and Depression NOS OR 7.02, 95%CI 2.07-23.82).

**Table 6:** Estimated percentages and Standard Errors (SE) for currently or lifetime receiving mental health care (all types) by type of depression among adolescents in Central Norway (weighted analyses)

	*Current mental health care*		*Life-time mental health care*	
	**Percentage**	**S.E**.	**Percentage**	**S.E**.

*Previous 2 months*				

No depression diagnosis	1.8	0.8	13.6	3.3

MDD	69.3	8.6	80.4	5.6

Dysthymia	24.1	3.7	57.4	4.2

MDD + Dysthymia	11.2	4.2	56.0	6.4

Depression NOS	9.7	2.8	39.7	12.0

Any Depressive Disorder	27.2	7.7	51.9	9.7

*Life-time*				

No depression diagnosis	0.2	0.	7.9	3.4

MDD	37.4	10.2	76.6	8.1

Dysthymia	17.4	9.0	50.6	13.7

MDD + Dysthymia	14.6	11.2	41.8	17.1

Depression NOS	8.6	4.0	37.6	9.8

Any Depressive Disorder	17.1	4.3	48.2	7.1

The following sources of care had been used by the adolescents (111 recorded contacts: 24 recorded twice): School health nurse 22.6%; school counseling 16.5%; teacher13.9%; child and adolescent psychiatry 13.9%; physicians at hospital or in general practice 13%; child protection services 9.6%; psychologists at the university 3.5%; "others" 4.3%; missing 2.2%. Twenty-four adolescents had received help more than once, while only three adolescents had received inpatient psychiatric service. In addition, four adolescents had been admitted to a somatic hospital for mental health reasons. Only one adolescent was currently on medication, and none had received any medication earlier because of a psychiatric problem. The most commonly reported reasons for contacts were: Problems with parents 23.4%; social problems 13.9%; affective problems 9.6%; school problems 8.7%; eating problems 11.3%; anxiety 7.8%; sexual abuse 4.3%; not specified 2.6%;. conduct problems 3.5%; and unknown/missing 14.8%.

## Discussion

### Prevalence of depression

Prevalence rates of various depressive disorders were estimated for community residing adolescents aged 14-16 years in Central Norway. The 2-month prevalence for Major Depressive Disorder (MDD) of 2.6% (CI 1.5- 4.4) was comparable to findings from Germany (3.4%) [9] and in the Netherlands (2.7%) [12], and in the UK (1.9%) in the same age group as in the present study [11], but lower than the prevalence of 5.0% in Switzerland [13] and 5.8% in Sweden [10], taking the confidence intervals of our findings into account. Our prevalence figure for current Dysthymia of 1.0% (CI 0.8-1.0) is in line with findings in earlier research [10,12,13].

It should be noted that Depression NOS rarely has been assessed in epidemiological surveys. While the prevalence of a current diagnosis was 6.3% (CI 3.7-10.3) and higher than the prevalence of minor depression of 2.6% (last month) among 15-24 year olds in the USA [36], and of 2.4% of subsyndromal depression in the Swedish study [10], it was much higher than the rate of 0.66% of current Depression NOS among 13-15-year olds in the UK study [11].

In the present study the lifetime prevalence for any depressive disorder was 23.0% (CI 18.1 -28.8), a finding in line with a rate 25% for any depressive disorder reported for somewhat older adolescents and young adults in the USA [36]. The relatively high rate of lifetime Dysthymia, rarely investigated in previous epidemiological surveys of general population samples, is consistent with the high prevalence of Dysthymia as reported among children in clinical studies [37].

However, our lifetime rate for MDD of 5.8% (CI 3.9-8.6) was lower compared to lifetime rates of 14.6% and 18.5%, respectively, among 15-18-year old adolescents in two epidemiological surveys in the USA [36,38], but similar to the 6.7% rate in the German study [9].

The reasons for the discrepancies in estimates across studies might be many fold. The inclusion of a younger age group of adolescents in the present study might have resulted in lower prevalence figures compared with studies including adolescents up to 17-18 years [10,12,38]. Further, different sampling methods might have affected the results. Here, both one- and two- wave studies are presented for comparisons, and all the studies include representative or total population samples. Most of the studies have both adolescents and parents as informants and use interviews based on DSM-IV, or DSM-III criteria including the use of impairment criteria. Lastly, the time frame used for definition of depressive episodes might have played a role. In the present study, current diagnoses had a relatively short time frame in order to aide recall, i.e. 2 months, while in other studies the time frame ranged from one month to one year [14]. However, since the episodes mostly have long duration our choice of time-frame is not likely to affect the validity of the comparisons to any great extent.

As a conclusion, this study adds to other studies the possibility of low rates of major depressive disorder among early adolescents in Central Norway, and further adds new information on the prevalence of Depression NOS.

### Gender differences

The girls faced a strongly increased risk for developing a MDD or current Dysthymia. In the present study the girls: boys ratio of current MDD was 11.8:1 and for Dysthymia 5.7:1; higher than what is usually found in other studies [14], but more in keeping with other European studies. This gender difference was attributable to a particularly low rate of MDD among boys. At present there is no ready explanation for the low rate of depression in adolescent boys reported in some studies in Europe [10,13]. Firstly, true prevalence might be low for severe depression among adolescent boys at this age. However, methodological constraints of the present study might have influenced our results. Due to our inclusion criteria inviting all individuals meeting screening criteria on the MFQ for subsequent interviews, only a limited number of boys were interviewed. This produced wide confidence intervals for prevalence rates underlining that our low rate of MDD in boys should be interpreted with caution. The lack of a significant gender difference in Depression NOS is at odds with a great body of findings regarding a gender difference of about 2:1 in sub-clinical depression, minor depression, and depressive symptoms [39], but this finding needs to be replicated. Because only a few boys were included, this might have prevented us from finding a true gender difference in the prevalence of Depression NOS. These considerations also pertain to the lack of gender difference in life-time Dysthymia. However, similar results on minor depression have also been reported in a national survey of adolescents and young adults aged 15 to 24 years in the USA [36].

### Characteristics of episodes

The duration of depressive episodes among adolescents was unusually long in the present study and some of them were still in an episode at the time of the study. The large variation in duration within each diagnostic subgroup of depression should also be noted. This aspect of adolescent depression has previously been investigated only to a limited extent. In the OADP study [40], the mean length of a MDD episode was about 6 months, shorter than in the present study. However, a duration of 8 months was found in a community sample of older adolescents [36] and 9 months among children in a clinical sample [37], both studies from the USA. In a Swedish study of somewhat older school adolescents, a one year duration was typical [10]. Longer episodes have also been associated with early onset MDD among adolescents and adults [40,41], and with depressive episodes among girls and women in community [42] and clinical samples [43]. Because our interview sample consisted predominantly of girls, this may have contributed to the extended episodes of MDD. Another explanation might be that depressive disorders last longer in recent cohorts or in Norway. However, this is at odds with the low 2-month prevalence of MDD in this study, and would, if true, suggest a particularly low incidence of MDD among adolescents in Norway. However, the duration of MDD in the present sample was shorter than the 17 month mean duration of MDD episodes in a clinical sample of both children and adolescents [44].

While the length of current Dysthymia in our study is comparable to a mean duration of 3.9 years in the study of clinical child and adolescent patients by Kovacs [37], past episodes of Dysthymia were shorter. Overall, the older the adolescents get, the longer a dysthymic episode appears to last.

In regard to the onset of depressive disorders, our data suggest that MDD episodes generally do not start before puberty, a finding in line with other studies both in the US [40] and in Europe [9], although this pertains to the recorded MDD episodes. Episodes of less severity, forexampel in the preschool age, were not recorded if the child later on experienced a more severe episode of depression. This also pertained to Depression NOS and Dysthymia starting in pre-puberty. A small group of children seemed to experience chronic Dysthymia, sometimes worsened and superimposed by MDD. Therefore, the assessment of depression in children should be carried out carefully by clinicians without the assumption that these disorders solely develop in adolescence.

### Functional levels

Our finding of significant reduced functional level in all depressive groups, especially among adolescents with MDD and "Double depression", is consistent with the findings of other community studies [35]. While it may seem contradictory that adolescents with current Dysthymia and Depression NOS had higher functioning levels than those with WPE or worst previous episodes. However, the worst previous episode may have been more impairing than the present one without any requirement of being the "worst" episode ever.

### Sociodemographics

The results of adjusted regression analyses regarding influences of socio-demographic showed that gender and not living with two biological parents were the strongest correlates of current severe depression. The higher prevalence of MDD in children of divorced parents is well documented [45]. Given that the rate of marital break-up is highest the first 10 years of a relationship, most divorces had preceded episodes of depression in the present sample. It is likely that negative psychosocial factors resulting from parental divorce such as decreased income, moving, custody conflicts, and a burdened situation with parents often lacking time and resources to monitor and support adolescents, may contribute to the development of depressive symptoms. Further, our findings of associations between severe depression and having parents in primary industry and living in inland areas might reflect the isolated lives adolescents sometimes experience in remote areas. Depression NOS was associated with living in the suburbs, but having parents belonging to the lower middle class lowered the risk.

Except for these findings, the present study did not obtain any associations between depression and SES, here defined as parental occupational status, nor with perceived economy. Inverse associations with SES have been shown in adult samples [46] and in epidemiological studies of adolescents with depressed mood [47] and with MDD [48]. However, when other factors have been controlled, as in the present population sample and in other studies of young people, these associations are weakened [49,50]. The relationships between depression groups and geography in the present study are difficult to interpret and uncertain due to wide confidence intervals. Lastly, small sample size might have increased the risk of type II errors; this could also be the case regarding the negative finding regarding ethnicity.

### Mental Health Care

Compared with other depressive groups, adolescents with MDD had more often received mental health care. Only one out of five of those with a current Dysthymia, and one in ten with a current diagnosis of "Double depression" were receiving some form of mental health care. Still, having any type of a depressive disorder increased the probability of receiving or having received mental health care. The findings also indicate that Depression NOS and Dysthymia among children and adolescents often are neglected. Even more noteworthy is that only 13.9% of current depressed adolescents were receiving help from specialist mental health services. These findings are in line with reports from an epidemiological study of children aged 8 to 10 in Norway, in which only 13.3% of those with emotional problems had been seen by a specialist [51]. More specifically, only 9.6% of the adolescents in the present study reported that affective symptoms were the main reason for previous contacts with a helping agency, while relationship problems accounted for almost half of the contacts. It is noteworthy that only one adolescent in the interview sample had ever been treated with medication because of depression, underscoring the lack of and need for specialist mental health care.

Despite a considerable growth in child psychiatric services in Norway during the last decades together with an increase of referrals because of depressed mood [52], specialist mental health services still reach only a minority of young people suffering from depression. Current knowledge about depressive disorders and their treatment in adolescence is also likely to be insufficient among primary care professionals and school health service providers.

### Strengths and limitations

This study had a high response rate of participating adolescents and their parents. A semi-structured interview performed by trained clinicians with adequate inter-rater agreement further supports the validity of the findings. Information on functional level adds to the validity of the study findings.

However, some limitations should also be considered. Retrospective data were used when assessing previous episodes of depressive disorders and might be influenced by recall bias, especially regarding the timing of symptoms. However, the use of parents as informants might have ensured a more detailed estimate of the onset of depressive symptoms, at least those occurring during early childhood [53], thus lending support to the validity and reliability of the duration data. Another limitation was that co-morbidity with depressive diagnoses, although assessed in the study, was not included in the analyses. Further, since other psychiatric disorders were not assessed separately, the obtained characteristics of depression might be unspecific and shared with other psychopathology. Our screening procedure resulted in few interviewed boys, making our obtained prevalence for them uncertain, and considered as preliminary. In the hinder sight we could have oversampled boys in the interview phase. We can, however, not totally disregard the possibility that the prevalence of depression is lower among European boys. On the whole, our confidence intervals were wide, and the results should be replicated in other studies before any definite conclusions can be drawn. Lastly, the study was confined to Central Norway, including both rural and urban areas. The adolescents in this part of the country appear to score only marginally lower than those in the remaining parts of Norway with respect to levels of depressive symptoms. Thus, it is assumed that the findings might be extended with caution to Norwegian adolescents in this age range, in particular for Norwegian adolescent girls.

## Conclusion

In the present study, a high rate of Depression NOS among adolescents with no gender difference was found, and future studies of the natural course and prognosis of Depression NOS are needed. Early onset of depressive episodes, with Depression NOS and Dysthymia starting in prepuberty, long duration, and low utilisation of specialist mental health services, emphasize an unmet need for proper diagnostic assessment, referrals and treatment of depressed adolescents.

## Competing interests

The authors declare that they have no competing interests.

## Authors' contributions

AMS, BL and LW contributed to the conceptualization of the study and were involved with the data analyses. AMS oversaw the collection of the data and drafted the manuscript. BL and LW contributed to the writing of the manuscript and all read and approved the final manuscript.

## References

[B1] Birmaher B Brent D Bernet W Bukstein O Walter H Benson RS Chrisman A Farchione T Greenhill L Hamilton J Practice parameter for the assessment and treatment of children and adolescents with depressive disorders J Am Acad Child Adolesc Psychiatry 2007 46 11 1503 1526 18049300 10.1097/chi.0b013e318145ae1c

[B2] Lewinsohn PM Rohde P Klein DN Seeley JR Natural course of adolescent major depressive disorder: I. Continuity into young adulthood J Am Acad Child Adolesc Psychiatry 1999 38 1 56 63 10.1097/00004583-199901000-00020 9893417

[B3] Keenan-Miller D Hammen CL Brennan PA Health outcomes related to early adolescent depression J Adolesc Health 2007 41 3 256 262 10.1016/j.jadohealth.2007.03.015 17707295 PMC2034364

[B4] Lewinsohn PM Rohde P Seeley JR Klein DN Gotlib IH Psychosocial functioning of young adults who have experienced and recovered from major depressive disorder during adolescence J Abnorm Psychol 2003 112 3 353 363 12943014 10.1037/0021-843x.112.3.353

[B5] Kashani JH McGee RO Clarkson SE Anderson JC Walton LA Williams S Silva PA Robins AJ Cytryn L McKnew DH Depression in a sample of 9-year-old children, Prevalence and associated characteristics Arch Gen Psychiatry 1983 40 11 1217 1223 6639292 10.1001/archpsyc.1983.01790100063009

[B6] Kim-Cohen J Caspi A Moffitt TE Harrington H Milne BJ Poulton R Prior juvenile diagnoses in adults with mental disorder: developmental follow-back of a prospective-longitudinal cohort Arch Gen Psychiatry 2003 60 7 709 717 10.1001/archpsyc.60.7.709 12860775

[B7] McGee R Feehan M Williams S Anderson J DSM-III disorders from age 11 to age 15 years J Am Acad Child Adolesc Psychiatry 1992 31 1 50 59 10.1097/00004583-199201000-00009 1537781

[B8] Costello J Mustillo S Erkanli A Keeler G Angold A Prevalence and development of psychiatric disorders in childhood and adolescence Arch Gen Psychiatry 2003 60 8 837 844 10.1001/archpsyc.60.8.837 12912767

[B9] Oldehinkel AJ Wittchen HU Schuster P Prevalence, 20-month incidence and outcome of unipolar depressive disorders in a community sample of adolescents Psychol Med 1999 29 3 655 668 10.1017/S0033291799008454 10405087

[B10] Olsson GI von Knorring AL Adolescent depression: prevalence in Swedish high-school students Acta Psychiatr Scand 1999 99 5 324 331 10353447 10.1111/j.1600-0447.1999.tb07237.x

[B11] Ford T Goodman R Meltzer H The British Child and Adolescent Mental Health Survey 1999: the prevalence of DSM-IV disorders J Am Acad Child Adolesc Psychiatry 2003 42 10 1203 1211 10.1097/00004583-200310000-00011 14560170

[B12] Verhulst FC van der Ende J Ferdinand RF Kasius MC The prevalence of DSM-III-R diagnoses in a national sample of Dutch adolescents Arch Gen Psychiatry 1997 54 4 329 336 9107149 10.1001/archpsyc.1997.01830160049008

[B13] Steinhausen H-C Winkler M Prevalence of affective disorders in children and adolescents: findings from the Zurich Epidemiological Studies Acta Psych Scand 2003 108 (suppl 418):20 23 10.1034/j.1600-0447.108.s418.5.x 12956809

[B14] Costello J Erkanli A Angold A Is there an epidemic of child or adolescent depression? J Child Psychology Psychiatry 2006 47 12 1263 1271 10.1111/j.1469-7610.2006.01682.x 17176381

[B15] Merikangas K Knight E Nolen-Hoeksema S, Hilt L The epidemiology of depression in adolescents Handbook of Depression in Adolescence 2009 New York London: Routledge 53 73

[B16] Essau CA Frequency and patterns of mental health services utilization among adolescents with anxiety and depressive disorders Depress Anxiety 2005 22 3 130 137 10.1002/da.20115 16175563

[B17] Wu P Hoven CW Bird HR Moore RE Cohen P Alegria M Dulcan MK Goodman SH Horwitz SM Lichtman JH Depressive and disruptive disorders and mental health service utilization in children and adolescents J Am Acad Child Adolesc Psychiatry 1999 38 9 1081 1090 discussion 1090-1082 10.1097/00004583-199909000-00010 10504806

[B18] Sund AM The development of depressive symptoms in early adolescence 2004 The Norwegian University of Science and Technology, Institute Of Neuroscience

[B19] Rossow I Grøholt B Wichstrøm L Intoxicants and suicidal behaviour among adolescents: changes in levels and associations from 1992 to 2002 Addiction 2005 100 1 79 88 10.1111/j.1360-0443.2005.00941.x 15598195

[B20] Kandel DB Davies M Epidemiology of Depressive Mood in Adolescents - an Empirical-Study Arch Gen Psychiatry 1982 39 10 1205 1212 7125850 10.1001/archpsyc.1982.04290100065011

[B21] Angold A Costello EJ Pickles EJ Winder F The development of a questionnaire for use in epidemiological studies of depression in children and adolescents 1987 London: Medical research Council

[B22] Sund AM Larsson B Wichstrom L Depressive symptoms among young Norwegian adolescents as measured by the Mood and Feelings Questionnaire (MFQ) Eur Child Adolesc Psychiatry 2001 10 4 222 229 10.1007/s007870170011 11794547

[B23] ISCO-1988 International Standard Classification of Occupation 1990 Geneva

[B24] Diagnostic and Statistical Manual of Mental Disorders 1994 4 Washington DC: American Psychiatric Association

[B25] Daviss WB Birmaher B Melhem NA Axelson DA Michaels SM Brent DA Criterion validity of the Mood and Feelings Questionnaire for depressive episodes in clinic and non-clinic subjects J Child Psychol Psychiatry 2006 47 9 927 934 10.1111/j.1469-7610.2006.01646.x 16930387

[B26] Wood A Kroll L Moore A Harrington R Properties of the mood and feelings questionnaire in adolescent psychiatric outpatients: a research note J Child Psychol Psychiatry 1995 36 2 327 334 10.1111/j.1469-7610.1995.tb01828.x 7759594

[B27] Cooper PJ Goodyer I A community study of depression in adolescent girls. I: Estimates of symptom and syndrome prevalence Br J Psychiatry 1993 163 369 374, 379-380 10.1192/bjp.163.3.369 8401968

[B28] Beck AT Ward CH Mendelson M Mock J Erbaugh J An inventory for measuring depression Arch Gen Psychiatry 1961 4 561 571 13688369 10.1001/archpsyc.1961.01710120031004

[B29] Kaufman J Birmaher B Brent D Rao U Flynn C Moreci P Williamson D Ryan N Schedule for Affective Disorders and Schizophrenia for School-Age Children-Present and Lifetime Version (K-SADS-PL): initial reliability and validity data J Am Acad Child Adolesc Psychiatry 1997 36 7 980 988 10.1097/00004583-199707000-00021 9204677

[B30] Shaffer D Gould MS Brasic J Ambrosini P Fisher P Bird H Aluwahlia S A children's global assessment scale (CGAS) Arch Gen Psychiatry 1983 40 11 1228 1231 6639293 10.1001/archpsyc.1983.01790100074010

[B31] Achenbach T Manual for the Child and Behaviour Checklist/4-18 and 1991 Profile 1991 Burlington VT: Dept of Psychiatry

[B32] Cantwell DP Lewinsohn PM Rohde P Seeley JR Correspondence between adolescent report and parent report of psychiatric diagnostic data J Am Acad Child Adolesc Psychiatry 1997 36 5 610 619 10.1097/00004583-199705000-00011 9136495

[B33] Diagnostic and Statistical Manual of Mental Disorders (DSM-IV-TR) 2000 American Psychiatric Association

[B34] Huber PJ The behavior of maximum likelihood estmates under nonstandard conditions The Fifth Berkeley Symposium on Mathamatical Statiotistics and Probality: 1967; Berkeley 1967 University of California Press 221 223

[B35] Goodman SH Schwab-Stone M Lahey BB Shaffer D Jensen PS Major depression and dysthymia in children and adolescents: discriminant validity and differential consequences in a community sample J Am Acad Child Adolesc Psychiatry 2000 39 6 761 770 10.1097/00004583-200006000-00015 10846311

[B36] Kessler RC Walters EE Epidemiology of DSM-III-R major depression and minor depression among adolescents and young adults in the National Comorbidity Survey Depress Anxiety 1998 7 1 3 14 10.1002/(SICI)1520-6394(1998)7:1<3::AID-DA2>3.0.CO;2-F 9592628

[B37] Kovacs M Obrosky DS Gatsonis C Richards C First-episode major depressive and dysthymic disorder in childhood: clinical and sociodemographic factors in recovery J Am Acad Child Adolesc Psychiatry 1997 36 6 777 784 10.1097/00004583-199706000-00014 9183132

[B38] Lewinsohn PM Hops H Roberts RE Seeley JR Andrews JA Adolescent psychopathology: I. Prevalence and incidence of depression and other DSM-III-R disorders in high school students J Abnorm Psychol 1993 102 1 133 144 8436689 10.1037//0021-843x.102.1.133

[B39] Wichstrom L The emergence of gender difference in depressed mood during adolescence: the role of intensified gender socialization Dev Psychol 1999 35 1 232 245 9923478

[B40] Lewinsohn PM Clarke GN Seeley JR Rohde P Major depression in community adolescents: age at onset, episode duration, and time to recurrence J Am Acad Child Adolesc Psychiatry 1997 33 6 809 818 10.1097/00004583-199407000-00006 7598758

[B41] Zisook S Rush AJ Lesser I Wisniewski SR Trivedi M Husain MM Balasubramani GK Alpert JE Fava M Preadult onset vs. adult onset of major depressive disorder: a replication study Acta Psychiatr Scand 2007 115 3 196 205 10.1111/j.1600-0447.2006.00868.x 17302619

[B42] Essau CA Lewinsohn PM Seeley JR Sasagawa S Gender differences in the developmental course of depression J Affect Disord 2010 127 1-3 185 190 10.1016/j.jad.2010.05.016 20573404 PMC3754427

[B43] Marcus SM Young EA Kerber KB Kornstein S Farabaugh AH Mitchell J Wisniewski SR Balasubramani GK Trivedi MH Rush AJ Gender differences in depression: findings from the STAR*D study J Affect Disord 2005 87 2-3 141 150 10.1016/j.jad.2004.09.008 15982748

[B44] Birmaher B Williamson DE Dahl RE Axelson DA Kaufman J Dorn LD Ryan ND Clinical presentation and course of depression in youth: does onset in childhood differ from onset in adolescence? J Am Acad Child Adolesc Psychiatry 2004 43 1 63 70 10.1097/00004583-200401000-00015 14691361

[B45] Amato PR Children of divorce in the 1990s: an update of the Amato and Keith (1991) meta-analysis J Fam Psychol 2001 15 3 355 370 11584788 10.1037//0893-3200.15.3.355

[B46] Lorant V Deliege D Eaton W Robert A Philippot P Ansseau M Socioeconomic inequalities in depression: a meta-analysis Am J Epidemiol 2003 157 2 98 112 10.1093/aje/kwf182 12522017

[B47] Lemstra M Neudorf C D'Arcy C Kunst A Warren LM Bennett NR A systematic review of depressed mood and anxiety by SES in youth aged 10-15 years Can J Public Health 2008 99 2 125 129 18457287 10.1007/BF03405459 PMC6975760

[B48] Roberts RE Roberts CR Chen YR Ethnocultural differences in prevalence of adolescent depression Am J Community Psychol 1997 25 1 95 110 9231998 10.1023/a:1024649925737

[B49] Sund AM Larsson B Wichstrom L Psychosocial correlates of depressive symptoms among 12-14-year-old Norwegian adolescents J Child Psychol Psychiatry 2003 44 4 588 597 10.1111/1469-7610.00147 12751850

[B50] Tracy M Zimmerman FJ Galea S McCauley E Stoep AV What explains the relation between family poverty and childhood depressive symptoms? J Psychiatr Res 2008 42 14 1163 1175 10.1016/j.jpsychires.2008.01.011 18308337 PMC2672881

[B51] Heiervang E Stormark KM Lundervold AJ Heimann M Goodman R Posserud MB Ullebo AK Plessen KJ Bjelland I Lie SA Psychiatric disorders in Norwegian 8- to 10-year-olds: an epidemiological survey of prevalence, risk factors, and service use J Am Acad Child Adolesc Psychiatry 2007 46 4 438 447 10.1097/chi.0b013e31803062bf 17420678

[B52] Reigstad B Jorgensen K Wichstrom L Changes in referrals to child and adolescent psychiatric services in Norway 1992--2001 Soc Psychiatry Psychiatr Epidemiol 2004 39 10 818 827 15669663 10.1007/s00127-004-0822-9

[B53] Angold A Erkanli A Costello EJ Rutter M Precision, reliability and accuracy in the dating of symptom onsets in child and adolescent psychopathology J Child Psychol Psychiatry 1996 37 6 657 664 10.1111/j.1469-7610.1996.tb01457.x 8894946

